# State of the art: Pregnancy in spinal muscular atrophy in the treatment era

**DOI:** 10.1177/22143602251370414

**Published:** 2025-09-12

**Authors:** Maggie C Walter, Bernert Günther, Blaschek Astrid, Deschauer Marcus, Günther René, Hiebeler Miriam, Hahn Andreas, Kamm Christoph, Kirschner Janbernd, Lochmüller Hanns, Löscher Wolfgang, Müller-Felber Wolfgang, Rudnik-Schöneborn Sabine, Schara-Schmidt Ulrike, Thiele Simone, Uzelac Zeljko, Vill Katharina, Weiler Markus, Hagenacker Tim

**Affiliations:** 1 Friedrich Baur Institute, Department of Neurology, 9183 LMU University Hospital, LMU Munich, Munich, Germany; 2 9169 Österreichische Muskelforschung, Wien, Österreich; 3 LMU University Hospital, Department of Pediatrics, Division of Pediatric Neurology, MUC iSPZ Hauner, Munich University Center for Children with Medical and Developmental Complexity, 9183 Dr von Hauner Children's Hospital, Munich, Germany; 4 Department of Neurology, Klinikum rechts der Isar, 9184 Technical University of Munich, School of Medicine and Health, Munich, Germany; 5 University Hospital Carl Gustav Carus Dresden, Technische Universität Dresden, Dresden, Germany; 6 Department of Pediatric Neurology, 9175 Justus-Liebig-University, Giessen, Germany; 7 Dept. of Neurology, 9187 University of Rostock, Rostock, Germany; 8 Dept. for Neuropediatrics and Muscle Disorders, 14879 Faculty of Medicine and Medical Center – University of Freiburg, Freiburg, Germany; 9 Children's Hospital of Eastern Ontario Research Institute, Division of Neurology, Department of Medicine, 113684 The Ottawa Hospital, Ottawa, Canada; 10 Brain and Mind Research Institute, 6363 University of Ottawa, Ottawa, Canada; 11 Department of Neurology, 27255 Medical University Innsbruck, Innsbruck, Austria; 12 Institute of Human Genetics, 27255 Medical University Innsbruck, Innsbruck, Austria; 13 Department of Neuropediatrics and Neuromuscular Centre for Children and Adolescents, Center for Translational Neuro- and Behavioral Sciences, Childreńs University Hospital Essen, 27170 University of Duisburg-Essen, Essen, Germany; 14 Department of Neurology, 199904 Ulm University, Ulm, Germany; 15 Department of Neurology, 9144 Heidelberg University Hospital, Heidelberg, Germany; 16 Department of Neurology, and Center for Translational Neuro- and Behavioral Sciences (C-TNBS), 123109 University Medicine Essen, Essen, Germany

**Keywords:** disease modifying treatment, nusinersen, parenthood, pregnancy, risdiplam, spinal muscular atrophy

## Abstract

An increasing number of adults with spinal muscular atrophy (SMA) wish to become parents. New disease-modifying therapies (DMT) have improved health outcomes and are expected to reduce disability in adults with SMA, but their current label prevents their use in pregnancy. While there is some information on pregnancy outcomes in the pre-DMT era, little has been published recently, and no ubiquitously accepted guidelines exist. Nonetheless, it is crucial to provide knowledgeable and open counselling, ideally in the context of treatments. Counseling for both adolescent and adult patients should include the subject of ‘reproductive choices’ when discussing the selection of DMTs for those considering parenthood. A multi-disciplinary team, including gynecologists and neurologists with expertise in neuromuscular disorders must closely monitor pregnant patients with SMA, preferably within disease registries, to detect potential complications early and ensure optimal treatment options are available. Real-world data in so far three patients with SMA showed a beneficial pregnancy outcome with nusinersen. It is anticipated that forthcoming real-world data will finally clarify the safety of administering Nusinersen during pregnancy, particularly in relation to child health and for preserving muscle function and preventing motor deterioration in the affected mother.

## Introduction

5q-associated spinal muscular atrophy (SMA) is an autosomal recessive disease, manifesting with predominantly proximal muscle weakness, accompanied by varying degrees of respiratory insufficiency. The incidence is 1:11,000 live births, and the prevalence amounts to 1–2/100,000 individuals, while the carrier frequency is 1:50.^1,2^ SMA is often perceived as a pediatric disease, although today most patients reach adulthood, so that at least one third of all SMA patients are now adults.^3^ In the pre-therapeutic era, the disease was classified into five phenotypes (SMA 0–4), which were characterized by age at onset and maximum motor function achieved. However, SMA represents a disease continuum, and a new classification categorizes phenotypes according to their best motor function as non-sitters, sitters and walkers.^4^

There are two almost identical forms of the *SMN* gene. *SMN1* is the gene primarily responsible to produce a functional SMN protein, *SMN2* is present in different copy numbers (2–8 copies) in the normal population and each copy can only produce approximately 10% of functional SMN protein compared to *SMN1* due to a C > T variant causing preferential splicing with the exclusion of exon 7. Therefore, the number of *SMN2* copies modifies the clinical expression of the disease; one *SMN1* copy results in SMA type 0,^5^ while only two *SMN2* copies are usually associated with a severe SMA1 phenotype.^6,7^ If more than 5 *SMN2* copies are present, SMA does not manifest despite the absence or defect of the *SMN1* gene since this is fully compensated by the number of *SMN2* copies. Understanding the molecular pathophysiology of SMA formed the basis for the development of current therapeutic approaches aiming to increase the amount of functional SMN protein, for example by including exon 7 in SMN2 transcripts or increasing *SMN2* gene expression or by direct gene addition of *SMN1*.^8,9^

In the light of now available disease modifying therapies (nusinersen became available in the US in 2016, onasemnogene abeparvovec in 2019, risdiplam in 2020, in Europe, each drug was approved one year later) the majority of SMA patients is now reaching adulthood and family planning is becoming increasingly relevant. A recent best practice guideline report from Schroth et al. found that 64% of patients rated pregnancy as very important, and 36% as somewhat important.^10^

Advances in treatment for SMA have allowed most individuals with the condition to attain reproductive age and consider pregnancy or fatherhood. Currently, research and guidelines concerning pregnancy in individuals with SMA remain scarce. These reports indicated increased caesarean section and premature labor rates. Although the likelihood of complications for mothers and offspring remained comparable in pregnant individuals with SMA to that of the general population, and fertility in women seemed not to be impaired.^11–14^ However, there was evidence for reduced fertility in male SMA patients,^15^ and reports about cryptorchidism in untreated male patients, eventually leading to fertility problems also in untreated male patients.^16,17^

So far, only three studies have highlighted experiences of pregnancies that occurred during Nusinersen treatment,^18–20^ demonstrating a paucity of data in the therapeutic era.

Therefore, the aims of this report are to recapitulate the literature concerning risks related to pregnancy for mothers and offspring, to summarize the current knowledge about the potential impact of new therapeutics now applied in SMA on pregnancy and fertility, and to give recommendations how to advice males and females regarding family planning, fertility, and pregnancy.

## Pregnancy outcomes in SMA in the pre-treatment era

To date, the available data remains insufficient for neurologists, pediatric neurologists and obstetricians to provide comprehensive guidance to their SMA patients on pregnancy and childbirth-related concerns. Most of the data currently accessible is anecdotal in nature and originates from smaller case series that focus on genetically diverse SMA populations prior to the advent of therapeutic interventions. A limited number of studies have demonstrated favorable outcomes for both mothers and offspring in most cases.^11–14,21^

Awater et al.^21^ updated the results of a German study including 25 genetically confirmed patients who had a total of 41 pregnancies. The number of early miscarriages was not increased, however obstetric complications were increased including a high frequency of caesarean deliveries (42,4%) and preterm deliveries (29,4%). Reduced lung function in pregnancy can be a major risk factor for mother and child, as was shown in a SMA type 2 patient who had a vital capacity (VC) of 19% and FEV1 of 17% in her only pregnancy. She developed hypercapnia and hypoxemia at 25 weeks gestation and had a stillbirth thereafter. Following the recommendations of an ENMC International Workshop, women with a VC of less than one liter are advised against pregnancy.^22^ Despite the fact that a possible influence of pregnancy on muscle weakness is difficult to assess in retrospect, persistent worsening of symptoms during or after pregnancy was experienced in about one third of SMA pregnancies.^21^

Research findings from Yim et al. indicate that similar complications have been observed in other studies,^23^ along with more urinary tract infections^24^ and worsening of pulmonary function.^11,25^

A 2017 cross-sectional study conducted in the USA involving 32 women with SMA (types 2 and 3) found that they had positive experiences and outcomes during pregnancy; however, the study advised that healthcare providers should exercise caution during counseling because most patients experienced intermittent or permanent declines in muscle strength and mobility after giving birth.^12^ An impressive 91% of individuals felt pleased with their decision to become parents and affirmed they would choose to do so again, and 85% indicated they were satisfied with the obstetric and anesthetic services provided to them. Among 35 pregnancies analyzed, researchers observed no notable variations in birth weight or APGAR scores; however, there was an increased incidence of preterm deliveries and cesarean sections compared to the reference population, particularly among patients with SMA type 2. A literature review by Abati et al.^11^ highlighted 100 pregnancies in 67 patients with SMA (N = 67; types 2, 3, and 4), reflecting a variety of disease characteristics. It was observed that whereas pregnant patients with SMA encountered difficulties, most reported favorable outcomes and experiences. In 42% of patients, researchers observed either a temporary or lasting decline in motor function. Notably, patients with SMA did not experience fertility issues or post-partum depression; however, those who used wheelchairs exhibited a decline in respiratory function during pregnancy. Anesthesia posed a greater risk for patients with already reduced lung function, leading to a higher likelihood of postoperative muscle weakness and respiratory insufficiency. In a study of 100 pregnancies, the frequency of fetal complications remained consistent with the reference population, but there was a significant rate of preterm births at week 36 and an increased incidence of cesarean sections, forceps, or vacuum deliveries, particularly among non-ambulant patients.^11^ Howarth^14^ described the successful management of a pregnancy in a SMA type 3 patient, with delivery by Caesarean section at 32 weeks, and a decline in muscle function post-delivery.

Potential effects of drugs used prior to the availability of SMN-enhancing drugs, such as salbutamol, were not considered in these studies. Today, salbutamol is no longer used by German or Austrian SMA patients (data from the German-Austrian SMA patient registry, www.sma-register.de). However, Salbutamol may still be used today in combination with SMN-enhancing drugs in other countries and therefore should be taken into account.

## Current situation and unmet need

Currently, adult patients with SMA have access to two unique drugs that modify disease progression: Nusinersen (approved 2016 by FDA, 2017 by EMA) and Risdiplam (approved 2020 by FDA, 2021 by EMA). As an intrathecally delivered antisense oligonucleotide, Nusinersen modifies the splicing of *SMN2* mRNA, thereby increasing the levels of the SMN2 protein. Although the clinical trials were conducted mainly in children,^26,27^ broad real-world evidence indicates that Nusinersen provides significant clinical benefits for adult patients with SMA, particularly in enhancing and maintaining motor function.^28–32^

Risdiplam acts as a small, orally bioavailable molecule that enhances splicing mechanisms, leading to increased synthesis of the functional SMN protein through the modulation of SMN2 splicing. Risdiplam demonstrated a notable clinical advantage in treating infantile spinal muscular atrophy (SMA) across three clinical trials: SUNFISH (focused on SMA types 2 and 3), FIREFISH (targeting SMA type 1), and JEWELFISH (involving patients with SMA types 2 and 3 previously treated with other DMTs). The benefits were less pronounced in cases of later-onset SMA.^33–36^

In 2020, EMA granted conditional, in 2022 full approval for the gene addition therapy onasemnogen abeparvovec in Europe, for patients with a confirmed mutations in the *SMN1* gene and a clinical diagnosis of SMA type 1, irrespective of their *SMN2* gene copy number. Additionally, it is approved for SMA patients with an *SMN1* mutation and up to three copies of the *SMN2* gene, regardless of the specific SMA classification.^37^ The 2024 European consensus statement on gene therapy for spinal muscular atrophy defined no specific age or weight limitations fined; however, the medication is exclusively administered to newborns and young children.^38,39^ Given that the potential risks associated with gene therapy increase in relation to the dosage of onasemnogen abeparvovec, and that this dosage correlates with a patient's weight and age, it is essential to consider treatment options for older and heavier individuals with great caution due to the scarcity of available data.

Individual patient outcomes with either therapeutic option depend predominantly on the degree of motor function preserved before the start of treatment, rather than on the type of SMA or the age of the patient.

Still, the experience with the use of Nusinersen in pregnant SMA patients is limited. Evidence from animal studies, as outlined in the information for healthcare professionals, revealed no signs of direct or indirect harmful effects on reproductive health. According to the information available for healthcare professionals, studies conducted in animals have not revealed any evidence of harmful effects, either directly or indirectly, associated with reproductive toxicity. Although there is only limited if any capacity of Nusinersen to traverse the blood-brain barrier, the information for healthcare professionals recommends avoiding Nusinersen in pregnant and breastfeeding individuals as a precautionary measure.^40^ It is currently recommended that after an interruption of Nusinersen therapy due to pregnancy and breastfeeding, treatment should be resumed according to the dosing schedule outlined in the Nusinersen Information for healthcare professionals. Pharmacokinetic data indicates that administering an extra dose for each missed maintenance dose is necessary to maintain consistent exposure levels in the cerebrospinal fluid.^41^ However, it should be reconsidered in the next years if the benefits of the treatment may outweigh the questionable risk of ongoing treatment with nusinersen during pregnancy.

Animal studies with Risdiplam have highlighted the risk of embryofetal toxicity; thus, an interruption of therapy for at least one month before attempting to conceive is recommended. Before starting Risdiplam therapy, healthcare providers must assess the pregnancy status of reproductive-capable patients, as the use of reliable contraception methods is generally necessary. SMA patients receiving Risdiplam should not breastfeed throughout the course of their treatment.^42^ Therefore, in patients who are planning a pregnancy, a switch to Nusinersen should be discussed.

Risdiplam also poses a potential risk to male fertility; healthcare professionals recommend a minimum hiatus of four months from the medication prior to attempting conception. It is essential to engage in discussions about fertility preservation strategies, such as sperm banking, with male patients of reproductive age before initiating treatment. However, as detailed in Magot et al.,^15^ a considerable amount of male patients are found to have azoospermia even prior to treatment, leaving sperm banking infeasible. In such cases, referral to a specialized fertility center may be proposed as part of the reproductive planning process.

So far, embryofetal toxicity and fertility problems are based on animal experiments; for human data, Roche is conducting an observational study for women with SMA who are or have been pregnant while taking Risdiplam (www.evrysdipregnancyregistry.com).

Recently, Erdler et al. presented a poster on fertility outcomes in three Risdiplam-treated male patients at the 2024 World Muscle Society Conference in Prague, resulting in the birth of two healthy infants.^43^ The first male patient received Risdiplam only two months prior to conception, the second patient was already one year on treatment until conception. The third patient was on Risdiplam for 2,5 years until accidental conception, and the pregnancy was terminated. No data was given if and how long the first two patients were planning of a pregnancy of their respective partners.

Until today, the unmet need in SMA patients with the desire to have children is high, and many aspects should be addressed during counselling - ongoing care by a multidisciplinary team (neuropediatricians/neurologists, geneticists, gynecologists, midwifes) before, during and after pregnancy and childbirth. Potential respiratory deterioration in severely affected SMA patients should be monitored closely and patients should also be evaluated regularly for respiratory function during pregnancy.

The topic of parenthood should become an integrated part of the patient consultation of adolescents and adults, and it is important to mention that a positive pregnancy outcome is as likely in SMA patients as in the general population. Additionally, counselling of the couple along with carrier testing of the partner should be recommended to patients with the wish to have children. In the US, only half of the patients with SMA received carrier testing of their spouses.^12^ As a future goal, development of specific information material regarding SMA and pregnancy for gynecologists would be warranted, along with close communication among specialists. Patients should be provided with a list of aids and possible support (e.g., requirements / conditions for parental assistance, options for pushing the baby carriage independently, exchange with other parents with SMA, etc.). The best approach to support patients in their parenting journey is a multidisciplinary approach involving rehabilitation physicians, midwives, and occupational therapists. In several countries, dedicated consultations for parenting with a disability are emerging and could be implemented within neuromuscular disease centers offering comprehensive guidance and support.

It is of utmost importance that SMA patients are regularly followed up in disease registries for gaining knowledge in a broader population of parenthood in SMA, in German speaking countries within the SMA patient registry (www.sma-register.de), which is a patient-based registry, and within the SMArtCARE registry (www.uniklinik-freiburg.de/smartcare.html), led and operated by neuromuscular specialists.

A recent brief report from Poland highlights a positive pregnancy outcome following the initiation of Nusinersen treatment in late pregnancy.^19^ During the 2^nd^ trimester of her pregnancy, a 21-year-old ambulatory patient was diagnosed with SMA type 3. Due to a deterioration in her ability to walk, the healthcare team commenced Nusinersen therapy in the 3^rd^ trimester, after securing individual case approval from the SMA treatment coordinating team, the Ministry of Health, and the National Health Fund in Poland. Although the outcome was positive, and the overall perspective of ongoing use of Nusinersen during pregnancy is promising, the current evidence remains insufficient to warrant alterations in the overarching guidelines regarding the use of Nusinersen during pregnancy.

Hiebeler and colleagues^20^ documented the case of a 33-year-old longtime wheelchair dependent patient with type 3 SMA, who got pregnant after 36 months on Nusinersen therapy. Nusinersen was last given in the 3^rd^ week of pregnancy. Following the confirmation of pregnancy, therapy was promptly discontinued. In the 34^th^ week of pregnancy, a healthy child was delivered by caesarean section. Following a brief period of nursing, Nusinersen treatment was resumed six weeks past the anticipated due date. Two years after the initial assessment, the patient conveyed that her motor functions had deteriorated and stabilized at a lower level than before pregnancy, even with the continuation of Nusinersen therapy.

In a case report authored by Schön and colleagues,^18^ a 36-year-old woman with SMA type 3 had Nusinersen therapy for twelve weeks into her pregnancy. Although the patient looked forward to having twins, one fetus did not progress due to growth retardation, a condition that was not considered to be a consequence of exposure to Nusinersen, as the second fetus developed normally. After the patient paused Nusinersen treatment, her motor function declined. Real-world evidence from adult patients clearly demonstrates that Nusinersen significantly benefits motor capabilities even in those with long-term disease and in more advanced stages.^28–32^

Another case study^44^ reported a 32-year-old patient with SMA type 3, who had been wheelchair-dependent since age 16 and who had received Nusinersen therapy, resulting in improvement of motor functions, for 21 months before conception. Due to severe scoliosis with spondylodesis at age 13, intrathecal application of Nusinersen had to be performed under CT guidance. After pregnancy was confirmed following the 8th Nusinersen injection, Nusinersen therapy was interrupted. Following successful delivery of a healthy child and lactation, Nusinersen therapy was resumed after twelve months. At baseline before resumption of therapy, no clinically meaningful deterioration of motor functions was observed (increase in RULM, slight decrease in HFMSE).

However, once treatment is discontinued, one should expect further progression that mirrors the natural trajectory of the disease. Relative to the pre-treatment data of pregnant SMA patients, the muscle function decline in reported by Hiebeler^20^ and Schön^18^ might be explained by the disease's natural progression without SMN restoration and, in part, to the well-documented accelerated decline that SMA patients experience during pregnancy.^11,13,18,20,25^

Still, prospective studies of pregnancies are required to answer the question of a possible exacerbation of SMA during or after pregnancy.

Recently, Finkel et al. prenatally treated a fetus predicted to have SMA1 with only two SMN2 copies by means of oral administration of risdiplam to the mother at a dose of 5 mg per day from the 32^nd^ week of pregnancy for six weeks until delivery, and the baby started risdiplam eight days after birth.^45^ No features of SMA, such as hypotonia, weakness, areflexia, or fasciculation have appeared to date. Notably, the child suffers from ventricular septal defect, which resolved, mildly reduced visual acuity with transient fixation nystagmus attributed to optic nerve hypoplasia in both eyes and mild right hemiparesis associated with left midbrain hypoplasia. Global developmental delay without regression has also been present in the infant to date. Genetic testing did not identify a second genetic disorder in genes associated with septo-optic dysplasia and neuroendocrine disorders. However, the congenital abnormalities were not seen by the investigators as related to the risdiplam exposure, since they were considered to have occurred early in fetal development, before exposure to risdiplam, and had also not been observed in prior animal studies.^45^ Still, results in this single case are not yet sufficient to support the consideration of prenatal risdiplam treatment for SMA identified in utero.

## Future plans and recommendation

It is essential for individuals with SMA to receive comprehensive information regarding the potential benefits and hazards associated with pregnancy as early as possible. Ideally, individuals should be counselled to contemplate starting a family while they still have a significant level of motor and respiratory abilities. While there are documented cases of patients with SMA type 2 successfully completing their pregnancies,^46,47^ it is crucial to plan for pregnancy at the earliest opportunity to minimize the risk of further complications. Still, the findings of Hiebeler et al.^20^ demonstrate that women with significant disabilities who are wheelchair dependent can still achieve successful pregnancies with favorable outcomes for both mother and child. Based on currently available data on animal research and real-world patient observations,^18–20^ in our opinion it is advisable to discuss with patients planning motherhood to only discontinue Nusinersen once pregnancy is confirmed, continuing this cessation throughout the pregnancy and breastfeeding period. Stopping Nusinersen treatment before conception as a precaution could lead to an extended non-treatment phase, increasing the risk of potentially irreversible motor deterioration. However, the final decision must always be made by the patient.

Due to the risk of potential toxicity in women, and a potential fertility risk in men, an interruption of therapy for at least one month before attempting to conceive is recommended in female patients on Risdiplam therapy, and for 4-months in male patients planning fatherhood as stated in the Risdiplam label and packet insert of the drug. However, this is debatable considering reports of reduced male fertility in the natural course of the disease.^15–17^

Consequently, the therapeutic management in SMA patients with the desire to have children in the nearer future should prioritize Nusinersen as the most suitable DMT, if intrathecal application is feasible. This topic should also be regularly addressed in the new counselling of newly diagnosed patients/parents, preferably before a specific DMT is selected.

Fertility does not seem to be impaired in women with SMA;^11–14^ however, diagnosis of SMA should not be an exclusion criterion regarding infertility treatment, since positive pregnancy outcomes are equally as likely as in the general population.

Table 1 provides a summary of the authors recommendations.

**Table 1.** Pregnancy and parenthood in SMA – recommendations.

• Provide knowledgeable and **open counselling**, ideally before making any treatment choices
• Counseling for both adolescent and adult patients must prioritize the subject of ‘**family planning’** when discussing the selection of disease-modifying therapies (DMT)
• **Genetic counseling** of the couple prior to pregnancy including the option to characterize the carrier status of the partner
• The patient should have access to a **multi-disciplinary team**, including geneticists, gynecologists, midwifes, neurologists and in case of adolescents pediatric neurologists with expertise in neuromuscular disorders
• **Pregnant SMA patients should be closely monitored**, preferably within disease registries, to detect potential complications early and ensure optimal treatment options
• Delivery should be planned in **specialized centers** with SMA experience
• Due to the potential risk of embryofetal toxicity in women, and a potential fertility risk in men, an **interruption of therapy for at least one month before attempting to conceive is recommended in female patients on Risdiplam therapy**, and for **4 months for male patients planning fatherhood**
• The therapeutic management in **SMA patients with the desire to have children** in the nearer future should **prioritize Nusinersen as the most suitable DMT**, if intrathecal application is feasible
• **Stopping Nusinersen treatment in women with SMA before conception** as a precautionary measure could lead to an extended non-treatment phase and increase the risk of potentially irreversible deterioration of motor functions
• Forthcoming real-world data will finally **clarify the safety of administering Nusinersen during pregnancy**, particularly in relation to child health and for preserving muscle function and preventing deterioration of motor functions in the affected mother

## Conclusion

An increasing number of patients with SMA highly prioritize their wish to become parents; considering this trend, it is crucial to provide knowledgeable and open counselling, ideally before making any treatment choices. Counseling for both adolescent and adult patients must prioritize the subject of ‘family planning’ when discussing the selection of disease-modifying therapies (DMT) for those wishing for parenthood. A multi-disciplinary team, including geneticists, gynecologists, midwifes, neurologists and in case of adolescents pediatric neurologists must closely monitor pregnant patients with SMA, preferably within disease registries, to detect potential complications early and ensure optimal treatment options are available, and delivery should be planned in specialized centers who have experience with the disease. Real-world data in so far three patients with SMA showed a beneficial pregnancy outcome with Nusinersen treatment during the 1^st^ and/or 3^rd^ trimester of pregnancy. It is anticipated that forthcoming additional real-world data will finally clarify the safety of administering Nusinersen during pregnancy, particularly in relation to child health and for preserving muscle function and preventing motor deterioration in the affected mother.
